# Response to Dr de Silva's letter

**DOI:** 10.1192/bjb.2021.98

**Published:** 2021-12

**Authors:** Gethin Morgan

**Affiliations:** Emeritus Professor of Mental Health, University of Bristol, Bristol, UK. Email: hilary.howard@blueyonder.co.uk

02 December 2020

Dr de Silva's wide-ranging review of suicide prediction strategies is very welcome: it includes a number of useful new ideas on how our predictive efforts can be taken forwards. I do not wish to take issue with any of them. My own paper, however, focuses more narrowly on two specific issues. The first highlights the way in which ongoing variation in severity of intent, usually due to the random and unpredictable occurrence of stress-related events, can confound our predictive efforts, and I suggest how we might circumvent this. The second aims to show that, in spite of attempts to dismiss its value, the assessment of suicidal ideation can have a useful role in the prediction process, provided it is applied correctly and used appropriately. My approach is in the nature of risk assessment, which has been criticised by some as being too dependent on negative issues. I hope I have shown that by helping to identify future hazards and so anticipate ways of dealing with them, this is not just a negative process. A capable clinician should surely be able to ensure that such assessment does not compromise the establishment of a good trusting relationship with the patient. My overall hope for the future of suicide prevention is that polarised views, in which different approaches are seen as either good or flawed, will not prevail. Good points from each and every approach can then be incorporated into an overall synthesis of preventive strategy that can be used in clinical practice.

